# Cardiotoxicity in low-to-moderate cardiovascular risk patients undergoing anti-HER2 therapy: a prospective cardiac magnetic resonance study

**DOI:** 10.2478/raon-2025-0043

**Published:** 2025-10-27

**Authors:** Sainan Cheng, Mei Deng, Linlin Qi, Fenglan Li, Jiaqi Chen, Shulei Cui, Yawen Wang, Jianing Liu, Yang Fan, Lizhi Xie, Jianwei Wang

**Affiliations:** 1 Department of Diagnostic Radiology, National Cancer Center/National Clinical Research Center for Cancer/Cancer Hospital, Chinese Academy of Medical Sciences and Peking Union Medical College, Beijing, China; 2 MR Research China, GE Healthcare, Beijing Economic-Technological Development Area, Beijing, China

**Keywords:** breast cancer, cardiotoxicity, cardiac magnetic resonance imaging, HER2, T1 mapping

## Abstract

**Background:**

The study aimed to investigate cardiotoxicity among individuals undergoing anti-human epidermal growth factor receptor 2 (HER2) therapy with a low-to-moderate risk of cardiovascular complications. Cardiac magnetic resonance (CMR) imaging was employed in the investigation.

**Patients and methods:**

HER2-positive breast cancer patients who underwent CMR examinations both before and during therapy (first follow-up: 3-5 months; second follow-up: 6-12 months) between January 2021 and December 2022 were prospectively included. Each patient was evaluated for the risk of cardiovascular toxicity.

**Results:**

Thirty-five HER2-positive breast cancer patients were included (48.86 ± 10.34 years). Eighty-nine percent of patients had low cardiovascular toxicity risk, and 11% had moderate cardiovascular toxicity risk. At follow-up CMR, nine (25.71%) patients developed cardiac dysfunction. At follow-up 1, there was a notable decrease in left ventricular ejection fraction, stroke volume index, cardiac output index, and absolute strain values, accompanied by higher T1 and T2 values as well as end-systolic volume index compared to baseline (p ≤ 0.002). At follow-up 2, the T1 and T2 values recovered to near baseline. The cardiac output index exhibited a continuous decline (p ≤ 0.022), while other variables were similar (p > 0.05). Furthermore, at follow-up 1, the T1 value displayed a marked increase in patients with 1-3 points in cardiovascular toxicity risk factors compared to those with no risk factors (p ≤ 0.043).

**Conclusions:**

It is common for patients with low-to-moderate cardiovascular risk to experience early cardiotoxicity during anti-HER2 therapy. T1 mapping was a valuable approach for quantifying the specific extent of subtle tissue damage.

## Introduction

Advancements in breast cancer therapy, especially with targeted treatments, have yielded promising results for individuals diagnosed with human epidermal growth factor receptor 2 (HER2)-positive breast cancer. While displaying remarkable anticancer effects, the frequent incidence of cardiotoxicity resulting from cancer treatments hampers their clinical application and could compromise the quality of life for cancer survivors.^1^ The European Society of Cardiology Guidelines on cardio-oncology recommend a comprehensive evaluation of cardiovascular toxicity risk prior to initiating anticancer therapy, along with diligent cardiovascular monitoring throughout the cancer treatment journey.^2^ Early detection of changes in myocardial structure and function plays a pivotal role in preventing treatment disruptions and maintaining cardiac function.^2–4^ However, in clinical practice, cardiac surveillance during anticancer therapies is often overlooked among breast cancer patients without high cardiovascular toxicity risk and among those who have received non-anthra-cycline-based HER2-targeted therapy. Hence, clarifying cardiac functional alterations in patients with low-to-moderate cardiovascular toxicity risk undergoing anti-HER2 therapy is crucial. This clarification can assist in devising more practical and cost-effective screening strategies.

Cardiac magnetic resonance (CMR) imaging plays a crucial role in monitoring cancer therapy-related cardiotoxicity in this scenario, as it enables non-invasive identification of myocardial pathologies such as diffuse fibrosis, oedema, and inflammation using tissue characterization techniques.^5^ While CMR studies have highlighted cardiac systolic function changes during anti-HER2 therapy, they have not assessed the cardiovascular toxicity risk of HER2-positive breast cancer patients.^6–9^ Data on CMR changes after anticancer therapy among HER2-positive patients with low-to-moderate cardiovascular toxicity risk are limited. Therefore, this study aims to explore alterations in myocardial structure and function among HER2-positive breast cancer patients presenting a low-to-moderate cardiovascular toxicity risk profile employing CMR.

## Patients and methods

### Study participants

During the period spanning from January 2021 to December 2022, we conducted prospective enrolment of patients diagnosed with HER2-positive breast cancer, all of whom were slated to undergo anti-HER2 therapy. The oncologist exercised discretion in managing anticancer therapy. The anti-HER2 therapy regimen comprises two categories: single-targeted therapy (trastuzumab) and dualtargeted therapy (trastuzumab plus pertuzumab). Within the dual-targeted therapy, there are further subdivisions: one includes anthracycline-based drugs (anthracycline chemotherapy followed by taxane plus dual-targeted therapy), and the other does not involve anthracycline-based drugs (taxane plus carboplatin plus dual-targeted therapy). The treatment lasts for a cycle of 21 days. The exclusion criteria comprised a history of prior anticancer therapy, pre-existing symptomatic cardiac conditions, contraindications to undergo CMR, and refusal to take part in the study.

Based on recent clinical study findings, most instances of cancer therapy-related cardiac dysfunction (CTRCD) manifest within the initial 6 months’ treatment. Therefore, our study was included three CMR examinations, conducted before therapy (baseline), in the early stages after therapy initiation (at 3~5 months for follow-up 1), and in the mid-to-late stages (at 6~12 months for followup 2). Those who underwent at least one followup CMR examination were eligible for inclusion in the study. The study obtained approval from the hospital’s research ethics committee (Approval No. NCC2020C-508) and all participants furnished written consent with full understanding.

According to the HFA-ICOS (Heart Failure Association–International Cardio-Oncology Society) cardiovascular toxicity risk stratification, a low-risk level is characterized by the absence of risk factors or the presence of one moderate risk factor, each of which is assigned 1 point, while a moderate-risk level is defined as having moderate risk factors totalling 2~4 points.^2^ The baseline 1-point cardiovascular toxicity risk factor including elevated baseline cardiac biomarkers with anthracycline chemotherapy, hypertension, chronic kidney disease, diabetes mellitus, anthracycline before HER2-targeted therapy, previous exposure to non-anthracycline chemotherapy, current smoker or significant smoking history, obesity (BMI > 30 kg/m^2^). The baseline 2-point cardiovascular toxicity risk factors including previous arrhythmia with HER2-targeted therapies, left ventricular ejection fraction (LVEF) 50-54%, elevated baseline cardiac biomarkers with HER2-targeted therapies, age 65-79 years, previous exposure to anthracycline, previous exposure to radiotherapy to left chest or mediastinum.

Symptomatic CTRCD indicates heart failure, a clinical syndrome characterized by primary symptoms like breathlessness, ankle swelling, and fatigue. Asymptomatic CTRCD is categorized into three levels: (1) mild, indicated by a LVEF of 50% or higher, alongside a notable decrease in global longitudinal strain (GLS) exceeding 15%; (2) moderate, indicated by a new decrease in LVEF of at least 10 percentage points resulting in an LVEF ranging from 40–49%, or a decrease in LVEF of less than 10 percentage points resulting in an LVEF ranging from 40–49%, accompanied by a new decline in GLS exceeding 15%; (3) severe, characterized by a new reduction in LVEF to below 40%.^2,10^

### CMR protocols and image analysis

Scanning was conducted utilizing a 3.0 T scanner (SIGNA Architect, GE Healthcare, Waukesha, WI, USA). We acquired images by employing breathholding techniques. Cine images were acquired in LV 2-chamber, 4-chamber, LV outflow tract and multisection stacks of short-axis views to perform LV function and volumes. Oedema evaluation was conducted using T2-weighted dual inversionrecovery imaging in the mid-LV short-axis orientation. For T1 mapping, a modified look-locker inversion-recovery sequence was employed, capturing images in a 3(3)3(3)5 heartbeat pattern. To conduct T2 mapping, a multi-echo fast spine echo sequence utilizing double inversion-recovery was utilized, featuring four distinct echo times: 10.8 ms, 32.4 ms, 54.0 ms, and 75.5 ms. Both T1 and T2 mapping sequences were acquired in the mid-LV short-axis orientation.

CMR parameters were calculated with the CVI42 software, developed by Circle Cardiovascular Imaging Inc. in Calgary, Canada, by a radiologist with eight years of expertise in CMR. The radiologist was blinded to patients’ clinical data during the analysis process. LV volumes parameters including LVEF, end-diastolic and end-systolic volumes, cardiac output, stroke volume and myocardial mass. Variables were adjusted for body surface area and expressed as indexes. Parameters (except for ejection fraction) were calibrated based on body surface area and presented as indices. We measured the T1 and T2 values at the mid-LV septum in the short-axis plane. GLS was mainly derived from LV 2-chamber and 4-chamber view. Global radial strain (GRS) and global circumferential strain (GCS) were calculated using short-axis imaging. For intraobserver reproducibility test, the same radiologist re-examined measurements in 12 randomly sampled patients after a one-month interval. For interobserver reproducibility test, a second observer, unaware of the patients’ clinical data, independently analysed scans from 12 randomly chosen patients.

### Statistical analysis

Continuous variables are displayed as means ± standard deviation. Categorical variables are displayed as frequencies and percentages. The analysis encompassed comparing measurements at two different time points, utilizing a paired test for continuous variables demonstrating normal distribution and employing the Wilcoxon signed-rank test for variables without a normal distribution. Comparison between groups (patients with and without risk factors and patients treated with anthracycline-based and non-anthracycline-based anti-HER2 therapies) were assessed using either the unpaired t-test or the Mann-Whitney U test. Statistical significance was established for p-values less than 0.05. Statistical analyses were conducted using SPSS Statistics version 26 (IBM) and Prism version 9.3.0 (GraphPad Software).

## Results

### Patient characteristics and cancer therapy-related cardiac dysfunction (CTRCD)

**FIGURE 1.**
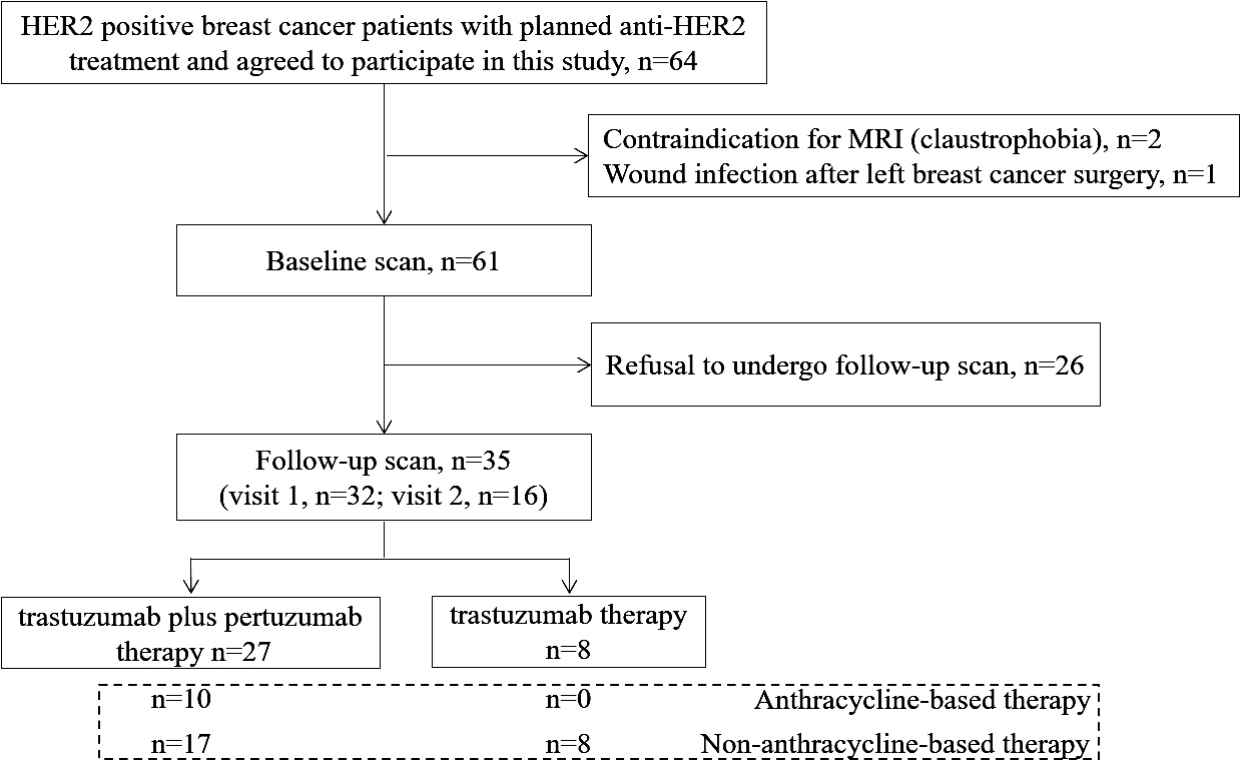
Study flow diagram. HER2 = human epidermal growth factor receptor 2

35 female patients diagnosed with HER2-positive breast cancer were ultimately included (mean age ± standard deviation, 48.86 years ± 10.34). Of the 35 patients, 17 (48%) received taxane plus carboplatin plus trastuzumab plus pertuzumab (TCbHP), 8 (23%) received taxane plus carboplatin plus trastuzumab (TCbH) and 10 (29%) received anthracycline chemotherapy followed by a taxane plus trastuzumab plus pertuzumab (AC/EC-THP) treatment (Figure 1). Five (14%) patients had hypertension, four were obese and two were above the age of 65 at baseline. All patients underwent an electrocardiogram examination before treatment, but for young patients with no prior medical history, cardiac biomarker tests were not performed. There were no apparent abnormalities in the patient’s baseline electrocardiogram. All patients were nonsmokers with no significant smoking history, and none had chronic kidney disease. 89% of the patients exhibited a low-risk level for cardiovascular toxicity, while 11% presented a moderate-risk level. Table 1 presents the foundational traits at the study’s outset.

**TABLE 1.** Clinical characteristics and treatment of the study participants

Variable	Value
No. of participants	35
Age, years*	48.86 ± 10.34
Height (cm)*	160.97 ± 4.20
Weight (kg)*	62.61 ± 8.36
Body surface area (m^2^)*	1.63 ± 0.12
Hypertension†	5 (14%)
Chronic kidney disease	0
Current smoker or significant smoking history	0
Obesity (BMI > 30 kg/m^2^) †	4 (11%)
Age 65–79 years†	2 (6%)
Diabetes	0
Hyperlipidaemia	0
Prior cardiovascular disease	0
HFA-ICOS baseline cardiovascular toxicity risk stratification†	
Moderate risk (moderate risk factors with a total of 2–3 points)	4 (11%)
Low risk	31 (89%)
No risk factor	18 (52%)
One moderate risk factor with a total of 1 point	13 (37%)
Therapies†	
HER2-targeted therapies without anthracycline	25 (71%)
Trastuzumab	8 (23%)
Trastuzumab and pertuzumab	17 (48%)
Anthracycline chemotherapy followed by a taxane plus trastuzumab plus pertuzumab	10 (29%)

* Data are given as means ± standard deviations.

† Data in parentheses are percentages.

1 BMI = body mass index; HFA-ICOS = Heart Failure AssociationInternational Cardio-Oncology Society; HER2 = human epidermal growth factor receptor 2

Thirty-two patients underwent cardiac MR scans at follow-up 1, with a mean time interval of 102.22 ± 20.58 days. Sixteen patients underwent cardiac MR scans at follow-up 2, with a mean time interval of 242.81 ± 68.29 days. Thirteen patients completed cardiac MR scans at both follow-up 1 and follow-up 2. For patients receiving both targeted therapy and anthracycline drugs, targeted therapy was administered sequentially after the completion of anthracycline treatment, with no instances of simultaneous usage.

We observed a significant increase in heart rate at follow-up 1 compared to baseline. However, no significant changes were detected in other vital signs, such as blood pressure or respiratory rate, during the same period.

A total of nine (25.71%) patients had experienced CTRCD during the follow-up CMR scans. Five of these patients showed no cardiovascular toxicity risk factors, while four of them showed 1-3 points risk factors.

One patient developed symptomatic CTRCD at follow-up 1, classified as New York Heart Association functional class III, with fatigue and dyspnoea as the primary symptoms. This was accompanied by a decrease in LVEF from 56% to 35%, a reduction in GLS from -11.18 to -9.48, a decrease in GRS from 18.65 to 15.78, a decrease in GCS from -12.76 to -11.42, an increased in native T1 value from 1239 msec to 1271 msec, and an increased in T2 value from 47.90 msec to 48.57 msec. The patient sought treatment at a hospital outside the local area, where she was treated with the traditional Chinese medicine for heart failure and also received antihypertensive therapy. Subsequently, the patient discontinued breast cancer treatment and did not return to our hospital for further follow-up.

**FIGURE 2.**
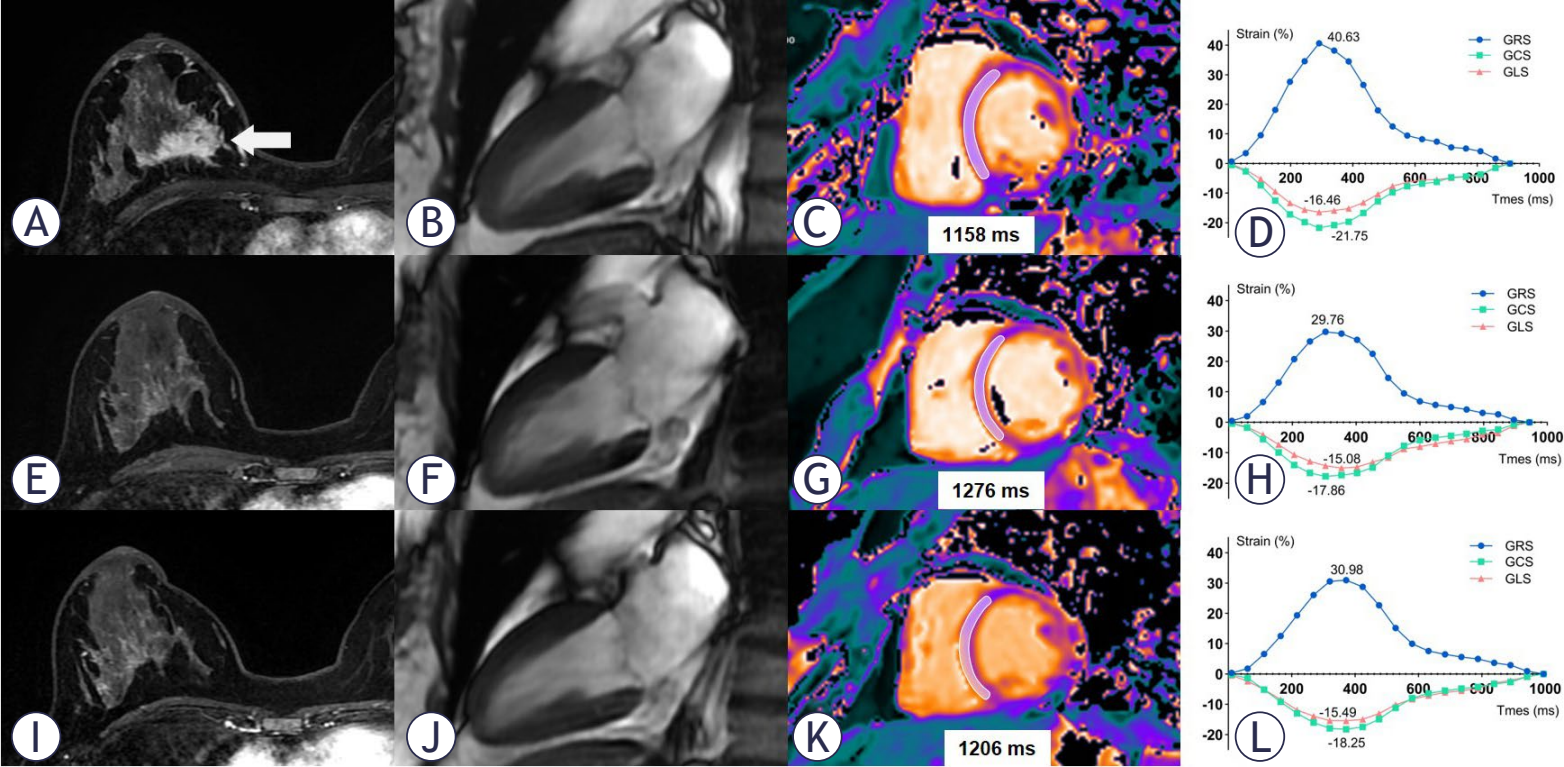
CMR imaging in a 44-year-old woman with human epidermal growth factor receptor 2 (HER2)-positive right-side breast cancer. The top row images **(A, B, C, D)** represent the baseline. The middle row images **(E, F, G, H)** correspond to the condition after 4 cycles of the TCbHP regimen (104 days after the baseline scan). The bottom row images **(I, J, K, L)** show the status after 9 cycles of the TCbHP regimen (203 days after the baseline scan). In the axial breast enhancement scan images **(A, E, I)**, a decrease in the tumour size of the right breast is observed (arrow). The diastolic phase images of left ventricular two-chamber plane **(B, F, J)** demonstrate a slight increase in left ventricular systolic volume. T1 mapping images **(C, G, I)** reveal an increase in T1 value in the septum of the mid short-axis slice during follow-up 1, followed by a decrease during follow-up 2. The global radial, circumferential and longitudinal strain (GRS, GCS and GLS) curves before and after TCbHP treatment are presented **(D, H, L)**. CMR = cardiac magnetic resonance; GCS = global circumferential strain; GLS = global longitudinal strain; GRS = global radial strain; HER2 = human epidermal growth factor receptor 2; TCbHP = taxane plus carboplatin plus trastuzumab plus pertuzumab

**FIGURE 3.**
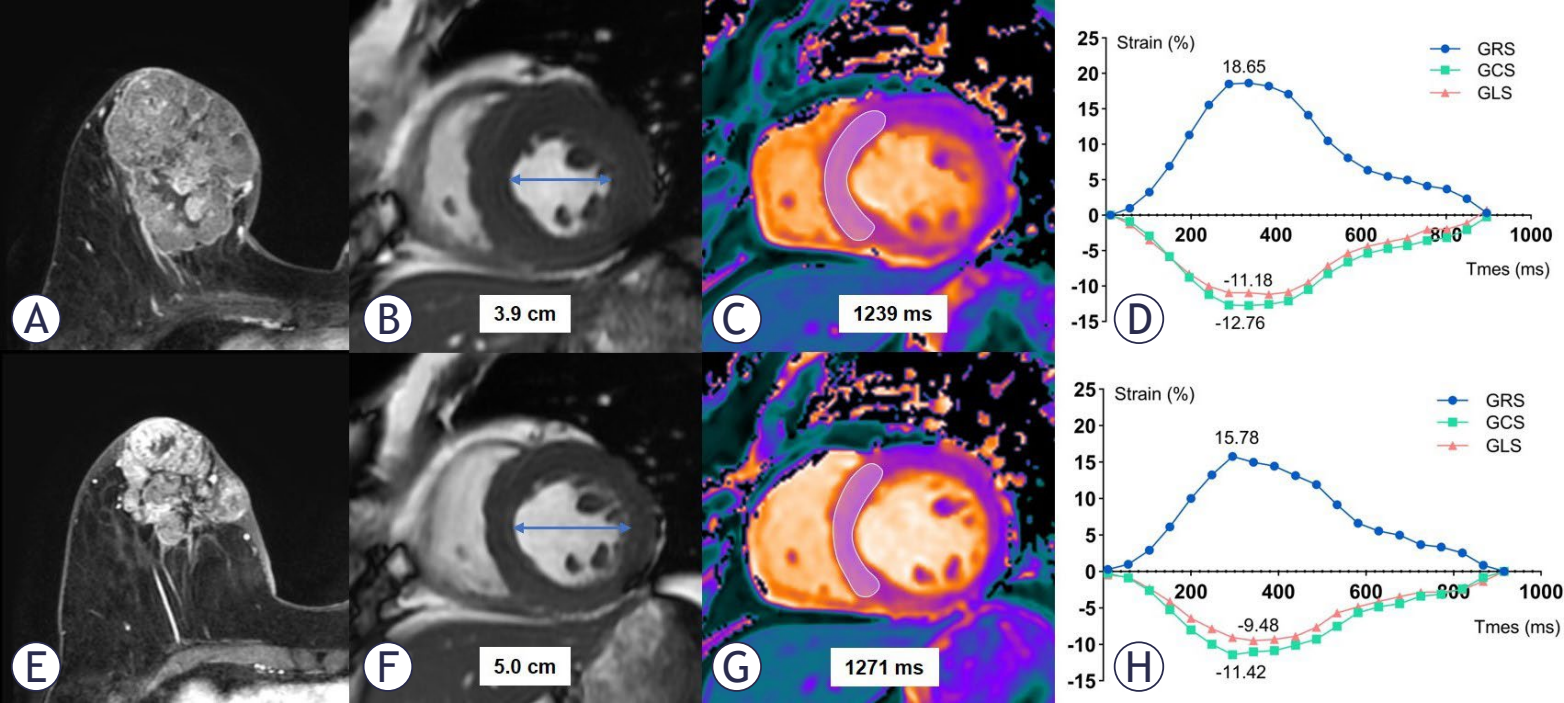
CMR imaging in a 68-year-old woman with HER2-positive right-side breast cancer. The upper images **(A, B, C, D)** depict the baseline condition. The lower images **(E, F, G, H)** represent the status after 4 cycles of the TCbHP regimen (106 days following the baseline scan). Axial breast enhancement scan images **(A, E)** exhibit a reduction in the tumour size of the right breast. Short-axis cine images reveal an enlargement in the left ventricular diastolic diameter **(B, F)**. T1 mapping images **(C, G)** reveal an increase in T1 value in the septum of the mid short-axis slice during the follow-up. Strain curves demonstrate a reduction in the absolute values of global radial, circumferential and longitudinal strain (GRS, GCS and GLS) before and after treatment **(D, H)**. CMR = cardiac magnetic resonance; GCS = global circumferential strain; GLS = global longitudinal strain; GRS = global radial strain; HER2 = human epidermal growth factor receptor 2; TCbHP = taxane plus carboplatin plus trastuzumab plus pertuzumab

Seven out of the nine patients exhibited mild asymptomatic CTRCD (six at follow-up 1 and one at follow-up 2), while one patient demonstrated moderate asymptomatic CTRCD at follow-up 2. Among the nine patients with CTRCD, three received anthracycline-based chemotherapy followed by taxane and dual-targeted therapy. Four received taxane in combination with carboplatin and dual-targeted therapy, while two were treated with taxane, carboplatin and trastuzumab. One patient with mild asymptomatic CTRCD had a history of hypertension for over 20 years and had been on long-term telmisartan therapy. None of the other patients received specific guideline-directed medical therapy for heart failure, such as ACE inhibitors, beta-blockers, or mineralocorticoid receptor antagonists, as recommended in the 2022 ESC cardio-oncology guidelines. Two representative clinical cases are shown in Figures 2 and 3.

### Differences in cardiac magnetic resonance (CMR) results before treatment and during the early stage after treatment

At follow-up 1, the LV systolic function showed a significant decrease compared to the baseline measurement, including reductions in LVEF (64.03% ± 4.16 *vs*. 57.34% ± 5.32; p < 0.001), stroke volume index (43.36 mL/m^2^ ± 8.12 *vs*. 37.10 mL/m^2^ ± 6.43; p < 0.001) and cardiac output index (3.23 L/min×m^2^ ± 0.58 *vs*. 2.91 L/min×m^2^ ± 0.49; p = 0.002). The decline in LV systolic function is primarily attributed to the increase in LV end-systolic volume (24.53 mL/m^2^ ± 6.17 *vs*. 28.26 mL/m^2^ ± 9.15; p = 0.001), with no significant changes observed in end-diastolic volume (p = 0.057). The LV mass index showed no significant statistical difference (p = 0.369).

We observed significantly decreases in absolute myocardial strain values: 33.59% ± 7.31 *vs*. 28.81% ± 6.37 for GRS, -18.86% ± 2.41 *vs*. -17.38% ± 2.31 for GCS, and -16.12% ± 1.55 *vs*. -15.09% ± 1.85 for GLS; all with a p-value of ≤ 0.001).

T2-weighted short-tau inversion-recovery imaging did not reveal apparent myocardial oedema. However, we noted significant increases in both T1 (1231.16 msec ± 46.49 *vs*. 1255.81 msec ± 45.23; p = 0.001) and T2 values (47.86 msec ± 2.17 *vs*. 49.43 msec ± 2.71; p = 0.001) (Table 2).

**TABLE 2.** Cardiac magnetic resonance (CMR) parameter results at baseline and follow-up

Variable	Follow-up 1 (n = 32)			Follow-up 2 (n = 16)		
	Baseline 1	Follow-up 1	P Value	Baseline 2	Follow-up 2	P Value
LV ejection fraction (%)	64.03 ± 4.16	57.34 ± 5.32	< 0.001	66.23 ± 4.19	57.45 ± 5.54	< 0.001
LV end-diastolic volume index (mL/m^2^)	68.23 ± 12.41	65.63 ± 12.97	> 0.057	68.20 ± 11.56	64.94 ± 14.01	0.275
LV end-systolic volume index (mL/m^2^)	24.53 ± 6.17	28.26 ± 9.15	0.001	22.91 ± 5.02	27.70 ± 7.60	0.002
LV mass index (g/m^2^)	37.17 (33.96, 43.69)	38.24 (34.40, 41.45)	0.369	39.23 ± 6.31	40.03 ± 6.78	0.261
Stroke volume index (mL/m^2^)	43.36 ± 8.12	37.10 ± 6.43	< 0.001	44.84 ± 8.12	39.46 (30.13, 42.17)	0.002
Cardiac output index (L/min×m^2^)	3.23 ± 0.58	2.91 ± 0.49	0.002	3.19 ± 0.38	2.65 ± 0.51	0.001
Global radial strain	33.59 ± 7.31	28.81 ± 6.37	0.001	37.16 ± 8.99	30.07 ± 6.18	< 0.001
Global circumferential strain	-18.86 ± 2.41	-17.38 ± 2.31	< 0.001	-19.93 ± 2.81	-17.23 ± 2.34	< 0.001
Global longitudinal strain	-16.12 ± 1.55	-15.09 ±1.85	0.001	-16.67 ± 1.28	-14.88 ± 1.60	0.004
T1 value (msec)	1231.16 46.49	1255.81 ± 45.23	0.001	1222.00 ± 40.89	1237.13 ± 35.19	0.051
T2 value (msec)	47.86 ± 2.17	49.43 ± 2.71	0.001	48.13 ± 2.15	48.84 ± 1.75	0.306
Heart rate during MR scan (bpm)	74.50 ± 11.11	79.47 ± 13.40	0.048	72.50 ± 9.68	72.69 ± 12.28	0.928

1 Data are given as means ± standard deviations or medians with interquartile ranges in parentheses. Baseline 1 and 2 were calculated separately for patients who underwent follow-up exams 1 and 2.

1 bpm = beats per minute; LV = left ventricular

### Differences in cardiac magnetic resonance (CMR) results before treatment and during the mid-to-late stage after treatment

**FIGURE 4.**
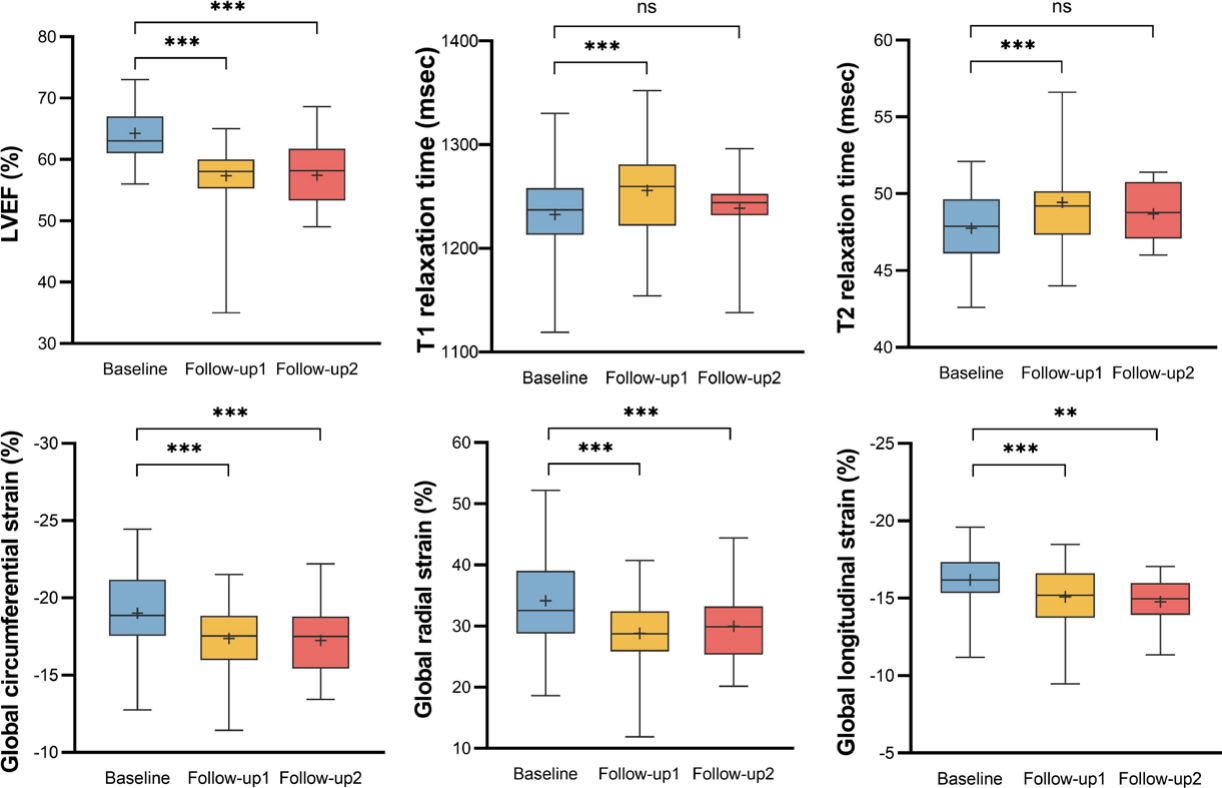
Box plot show changes in cardiac magnetic resonance (CMR) variables between the baseline and follow-up CMR examinations. The results of CMR variables at baseline (n = 35), follow-up 1 (n = 32) and follow-up 2 (n = 16) were shown in blue, yellow and red box plot. Left ventricular ejection fraction (LVEF) decreased significantly at follow-up 1 and follow-up 2. T1 and T2 value increased significantly at follow-up 1, but recovered to a level not significantly different from the baseline. Global longitudinal strain (GLS), global radial strain (GRS) and global circumferential strain (GCS) decreased significantly at follow-up 1 and follow-up 2.

Similarly to the aforementioned findings, there were significant decreases observed in LVEF, stroke volume index, cardiac output index, and absolute strain parameter values from baseline to follow-up 2 (p < 0.05). Conversely, the LV end-systolic volume index showed a notable increase (p = 0.002). Noteworthy, no substantial changes were detected in T1 and T2 values (p > 0.05) (Table 2, Figure 4).

### Differences in cardiac magnetic resonance (CMR) results between two subsequent follow-up scans

The average time span between the two subsequent follow-up CMR scans was 136.92 ± 67.70 days. A notable decrease in cardiac output index was observed (3.01 L/min×m^2^ ± 0.47 *vs*. 2.60 L/min×m^2^ ± 0.54; p = 0.022). No statistically notable differences were detected in the remaining variables (p > 0.05) (Table 3).

**TABLE 3.** Cardiac magnetic resonance (CMR) parameter results between follow-up 1 and follow-up 2 (n = 13)

Variable	Follow-up 1	Follow-up 2	P Value
LV ejection fraction (%)	58.45 ± 4.44	57.76 ± 5.53	0.545
LV end-diastolic volume index (mL/m^2^)	66.33 ± 11.13	62.32 ± 14.02	0.055
LV end-systolic volume index (mL/m^2^)	27.59 ± 6.20	26.35 ± 7.46	0.294
LV mass index (g/m^2^)	38.07 (36.22, 44.09)	39.13 (35.77, 43.31)	0.650
Stroke volume index (mL/m^2^)	39.37 (35.27, 43.55)	39.44 (27.44, 40.83)	0.101
Cardiac output index (L/min×m^2^)	3.01 ± 0.47	2.60 ± 0.54	0.022
Global radial strain	28.69 ± 8.12	29.70 ± 6.48	0.670
Global circumferential strain	-17.70 ± 2.52	-17.12 ± 2.30	0.172
Global longitudinal strain	-15.01 ± 1.46	-14.74 ±1.70	0.647
T1 value (msec)	1245.31 ± 22.46	1236.77 ± 40.36	0.486
T2 value (msec)	49.67 ± 2.19	49.11 ± 2.12	0.465
Heart rate during MR scan (bpm)	78.38 ± 14.67	73.92 ± 13.09	0.189

1 Data are given as means ± standard deviations or medians with interquartile ranges in parentheses.

1 bpm = beats per minute; CMR = cardiac magnetic resonance; LV = left ventricular

### Differences in cardiac magnetic resonance (CMR) results between patients with and without risk factors at the early stage after treatment

**FIGURE 5.**
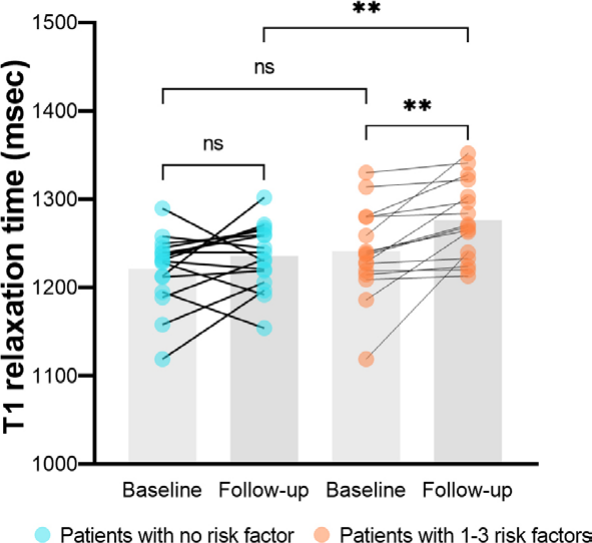
The comparison of T1 value between the baseline and follow-up cardiac magnetic resonance (CMR) examinations. Bars represent median T1 value. Blue dots represent human epidermal growth factor receptor 2 (HER2)-positive breast cancer patients with no cardiovascular toxicity risk factor and orange dots represent patients with 1–3 risk factors.

Thirty-two patients completed cardiac MR scans at follow-up 1, 16 of whom had no cardiovascular toxicity risk factors and 16 of whom had 1-3 risk factors. Patients presenting with 1-3 risk factors exhibited significantly higher LV mass index (37.07 g/m^2^ ± 6.68 *vs*. 43.03 g/m^2^ ± 8.86, p = 0.040) and T1 value (1235.06 ± 36.77 *vs*. 1276.56 msec ± 44.25, p = 0.007) (Figure 5). However, no statistically significant disparity in LV mass index or T1 value was observed between these two groups at baseline. The two groups also had similar LV ejection fraction, volume index, cardiac output index, strain parameters and T2 values (p > 0.05) (Table 4).

**TABLE 4.** Differences in CMR parameters at follow-up 1 between patients with and without baseline risk factors

Variable	Patients with no risk factor (n = 16)	Patients with 1-3 risk factors (n = 16)	P Value
LV ejection fraction (%)	57.92 ± 3.43	56.75 ± 6.79	0.543
LV end-diastolic volume index (mL/m^2^)	63.88 ± 10.89	67.39 ± 14.92	0.454
LV end-systolic volume index (mL/m^2^)	26.89 ± 5.66	29.63 ± 11.70	0.406
LV mass index (g/m^2^)	37.07 ± 6.68	43.03 ± 8.86	0.040
Stroke volume index (mL/m^2^)	36.60 ± 6.67	37.61 ± 6.36	0.665
Cardiac output index (L/min×m^2^)	2.84 ± 0.48	2.99 ± 0.49	0.387
Global radial strain	30.56 ± 5.40	27.07 ± 6.94	0.123
Global circumferential strain	-17.94 ± 2.16	-16.82 ± 2.37	0.174
Global longitudinal strain	-15.11 ± 1.85	-15.06 ± 1.90	0.938
T1 value (msec)	1235.06 ± 36.77	1276.56 ± 44.25	0.007
T2 value (msec)	48.95 ± 2.28	49.94 ± 3.07	0.318
Heart rate during MR scan (bpm)	77.56 ± 11.28	81.38 ± 15.37	0.430

1 Data are given as means ± standard deviations or medians with interquartile ranges in parentheses.

1 CMR = cardiac magnetic resonance; LV = left ventricular

### Differences in cardiac magnetic resonance (CMR) results between patients with and without anthracycline therapy at the early stage after treatment

Ten patients were taking anthracycline-based therapy and 22 patients were taking non-anthra-cycline-based therapy at follow-up 1. The group treated with anthracycline showed a notably elevated T1 value (1280.90 msec ± 43.10 *vs*. 1244.41 msec ± 42.29, p = 0.032). At baseline, the T1 value did not show any significant variance between the two groups. The two groups were similar in all other variables (p > 0.05) (Table 5).

**TABLE 5.** CMR parameter results between patients treated with anthracycline-based and non-anthracycline-based antihuman epidermal growth factor receptor 2 (HER2) therapies at follow-up 1

Variable	Anthracycline-based therapy (n = 10)	Non-anthracycline-based therapy (n = 22)	P Value
LV ejection fraction (%)	57.01 ± 3.68	57.49 ± 5.99	0.816
LV end-diastolic volume index (mL/m^2^)	67.51 ± 12.83	64.78 ± 13.24	0.590
LV end-systolic volume index (mL/m^2^)	28.88 ± 6.01	27.98 ± 10.38	0.800
LV mass index (g/m^2^)	41.76 ± 7.91	39.28 ± 8.53	0.442
Stroke volume index (mL/m^2^)	38.58 ± 7.78	36.43 ± 5.80	0.389
Cardiac output index (L/min×m^2^)	3.13 ± 0.49	2.82 ± 0.46	0.094
Global radial strain	26.73 ± 6.62	29.76 ± 6.16	0.218
Global circumferential strain	-17.09 ± 1.92	-17.52 ± 2.49	0.637
Global longitudinal strain	-15.36 (-16.56, -13.74)	-15.07 ± 2.05	1.000
T1 value (msec)	1280.90 ± 43.10	1244.41 ± 42.29	0.032
T2 value (msec)	49.65 (47.06, 53.10)	49.04 ± 2.23	0.231
Heart rate during MR scan (bpm)	84.20 ± 18.44	77.32 ± 10.20	0.182

1 Data are given as means ± standard deviations or medians with interquartile ranges in parentheses.

1 CMR = cardiac magnetic resonance; LV = left ventricular

## Discussion

In this research, our aim was to explore cardiotoxicity among individuals undergoing anti-HER2 therapy with a low-to-moderate risk of cardiovascular complications. The main findings are as follows: (1) Subclinical CTRCD was frequently observed in low-to-moderate cardiovascular toxicity risk patients with anti-HER2 therapy. (2) CTRCD was detected as early as the third month of anticancer therapy and persisted for at least approximately eight months. (3) After anticancer therapy, patients with 1–3 cardiovascular toxicity risk points, including those who underwent anthra-cycline-based therapy, exhibited higher T1 values compared to those without such risk factors, while showing no notable differences in LVEF or myocardial strain values.

Breast cancer patients categorized with low-to-moderate cardiovascular toxicity risk demonstrate an extended life expectancy relative to those with high cardiovascular toxicity risk, potentially allowing for the consideration of more aggressive targeted therapy and chemotherapy interventions.^10^ In our study, 89% of breast cancer patients were at low cardiovascular toxicity risk, and 52% had no risk factors. As demonstrated in Table 4, the presence of risk factors did not appear to significantly influence CMR parameters at follow-up 1. Patients with 1-3 risk factors exhibited higher LV mass index and native T1 values, whereas other parameters, such as LV ejection fraction, volume index, cardiac output index, strain parameters and T2 values, remained comparable between groups. These findings suggest that monitoring cardiac function during anticancer therapy is equally important even in patients with low cardiovascular toxicity risk.

In our study, 25.71% of patients developed CTRCD during treatment, as monitored through CMR examinations. Among the CTRCD patients, more than half had no risk factors before anticancer treatment. Despite the relatively low-to-moderate risk profile of our study population, the determined rate of CTRCD aligned with the ranges in earlier studies^11,12^ and was similar to a recent result.^13^ Thus, this is a critical group to monitor for cardiac toxicity during anticancer therapy. Regular evaluation of LV function, utilizing measurements of LVEF and GLS, is advised before starting HER2-targeted therapy and should continue every three months during the treatment monitoring period.^2,14^ In the case of asymptomatic mild CTRCD within this patient cohort, the guidelines propose the consideration of cardioprotective therapy (angiotensin-converting enzyme inhibitors, angiotensin receptor blockers and beta-adrenergic blockers). Additionally, it’s recommended to continue HER2-targeted treatment.^2,14–16^ However, cardiac surveillance is not always strictly performed in real-world clinical practice. In a clinical study encompassing 4,325 patients, it was observed that just 46.2% of breast cancer patients receiving trastuzumab underwent the suggested cardiac surveillance.^17^ Among low cardiovascular toxicity risk patients, the frequency of cardiac surveillance might be even less frequent in actual practice. For low cardiovascular toxicity risk patients, monitoring cardiac function during anticancer therapy remains equally important, but perhaps the monitoring frequency could be appropriately reduced.

In our study, cardiac dysfunction was observed at the third month of anticancer therapy, and this significant decline persisted into the eighth month. Cardiac motion function did not change significantly between the third and eighth months.

Notably, there was a substantial rise in T1 and T2 values noted at the early stage after treatment, but by the mid-to-late stage after treatment, these values had reverted to a level that did not show significant variance compared to the baseline. In essence, cardiotoxicity manifested as early as the third month of anticancer therapy, but for the majority of patients, its progression did not continue beyond approximately the eighth month. Our study aligns with findings from prior research. Houbois *et al.’s* findings suggest a decrease in both LVEF and strain values over the follow-up duration, with the lowest point reached at three months into trastuzumab therapy.^7^ At both 6 and 12 months, Gong *et al*. observed noteworthy declines in systolic function; however, these disparities ceased to be significant by the 18-month mark.^9^ Recent analyses on real-world data demonstrated that the time of onset of HER2 inhibitor-related cardiotoxicity was 80.50~103.00 days.^18,19^ This finding holds significance, indicating that among individuals diagnosed with HER2-positive breast cancer and presenting mild-to-moderate cardiovascular toxicity risk factors, conducting early and less frequent cardiotoxicity screenings could potentially improve cost-effectiveness.

Another significant observation from this study was that HER2-positive breast cancer patients with 1-3 cardiovascular toxicity risk factors displayed a minor yet statistically significant elevation in native T1 value following anticancer therapy, in comparison to those without any risk factors. However, there were no notable variations found in terms of statistical significance concerning LV ejection fraction or myocardial strain values. Typically, a rise in native T1 values correlates with myocardial conditions such as oedema, inflammation, and fibrosis^20^, which is able to reflect subtle myocardial histological changes. A previous clinical study has demonstrated that early cardiac changes, there was a progressive increase in native T1 and T2 values. Subsequently, as treatment progressed, there was a normalization of T2 time, while noncontrast T1 values remained persistently elevated, indicating the presence of myocardial fibrosis.21 The detection rate of cardiac abnormalities after cancer therapy was 87% based on T1 and T2 mapping. In comparison, abnormalities based on GLS and LVEF were found in 71% and 66% of patients respectively.^21^ Therefore, in identifying myocardial alterations among HER2-positive breast cancer patients following anticancer therapy, T1 mapping is a favoured method for precisely measuring the particular degree of tissue damage. This specific damage might not be easily identified through traditional cine sequences or strain analysis.

During the follow-up period, we noticed a notable increase in native T1 values among patients who underwent anthracycline-based therapy compared to those who underwent non-anthracycline-based therapy. This difference may be attributed to the potential of anthracycline chemotherapy to induce acute, subacute, and chronic myocellular injuries. Our findings align with a previous study, demonstrating increased T1 value in individuals with prior anthracycline chemotherapy exposure.^22^

There were several limitations acknowledged in this study. Firstly, the sample size was comparatively limited. This prospective study aimed to compare CMR parameters before and after anticancer treatment, illustrating the temporal changes in these parameters over time. Consequently, only patients with at least two CMR scans were included, underscoring the necessity for a study with a larger sample size. Secondly, the follow-up duration for CMR monitoring was short, which hindered the assessment of the enduring cardiac repercussions of anti-HER2 therapy. This area requires further investigation in future research. Thirdly, cardiac biomarkers included in patients’ haematological tests were not used for cardiovascular toxicity risk stratification prior to treatment, which may have introduced some bias. However, most patients in our cohort did not receive anthra-cycline-based chemotherapy and were relatively young, without a history or symptoms of cardiovascular disease, making abnormal biomarker levels unlikely. Therefore, the impact of this limitation was expected to be minimal, although it would be addressed more thoroughly in future studies.

In conclusion, asymptomatic cardiac dysfunction was common among HER2-positive breast cancer patients with low-moderate cardiovascular toxicity risk after cancer therapy. This subgroup of patients can potentially benefit from early postanticancer therapy monitoring at longer intervals, thus enhancing cost-effectiveness. T1 mapping is a valuable approach for quantifying the specific extent of tissue damage, particularly in the presence of subtle tissue changes. There’s a need for studies that encompass larger cohorts and extended follow-up durations.
